# Comparison of the Antioxidant and Cytoprotective Properties of Extracts from Different Cultivars of *Cornus mas* L.

**DOI:** 10.3390/ijms25105495

**Published:** 2024-05-17

**Authors:** Tadeusz Pomianek, Martyna Zagórska-Dziok, Bartosz Skóra, Aleksandra Ziemlewska, Zofia Nizioł-Łukaszewska, Magdalena Wójciak, Ireneusz Sowa, Konrad A. Szychowski

**Affiliations:** 1 Department of Management, Faculty of Administration and Social Sciences, University of Information Technology and Management in Rzeszow, Sucharskiego 2, 35-225 Rzeszow, Poland; tpomianek@wsiz.edu.pl; 2 Department of Technology of Cosmetic and Pharmaceutical Products, Medical College, University of Information Technology and Management in Rzeszow, Sucharskiego 2, 35-225 Rzeszow, Poland; mzagorska@wsiz.edu.pl (M.Z.-D.); aziemlewska@wsiz.edu.pl (A.Z.); zniziol@wsiz.edu.pl (Z.N.-Ł.); 3 Department of Biotechnology and Cell Biology, Medical College, University of Information Technology and Management in Rzeszow, Sucharskiego 2, 35-225 Rzeszow, Poland; bskora@wsiz.edu.pl; 4 Department of Analytical Chemistry, Medical University of Lublin, Aleje Raclawickie 1, 20-059 Lublin, Poland; magdalena.wojciak@umlub.pl (M.W.); ireneusz.sowa@umlub.pl (I.S.)

**Keywords:** cornelian cherry, dogwood, plant extract, PPARγ, skin cells

## Abstract

*Cornus mas* L. is a rich source of vitamin C and polyphenols. Due to their health-benefit properties, *C. mas* L. extracts have been used in, e.g., dermatology and cosmetology, and as a food supplement. Peroxisome proliferator–activated receptor gamma (PPARγ) and its co-activator (PGC-1α) are now suspected to be the main target of active substances from *C*. *mass* extracts, especially polyphenols. Moreover, the PPARγ pathway is involved in the development of different diseases, such as type 2 diabetes mellitus (DM2), cancers, skin irritation, and inflammation. Therefore, the aim of the present study was to evaluate the PPARγ pathway activation by the most popular water and ethanol extracts from specific *C. mas* L. cultivars in an in vitro model of the human normal fibroblast (BJ) cell line. We analyzed the content of biologically active compounds in the extracts using the UPLC-DAD-MS technique and revealed the presence of many polyphenols, including gallic, quinic, protocatechuic, chlorogenic, and ellagic acids as well as iridoids, with loganic acid being the predominant component. In addition, the extracts contained cyanidin 3-*O*-galactoside, pelargonidin 3-*O*-glucoside, and quercetin 3-glucuronide. The water–ethanol dark red extract (DRE) showed the strongest antioxidant activity. Cytotoxicity was assessed in a normal skin cell line, and positive effects of all the extracts with concentrations ranging from 10 to 1000 µg/mL on the cells were shown. Our data show that the studied extracts activate the PPARγ/PGC-1α molecular pathway in BJ cells and, through this mechanism, initiate antioxidant response. Moreover, the activation of this molecular pathway may increase insulin sensitivity in DM2 and reduce skin irritation.

## 1. Introduction

*Cornus mas* L., also known as Cornelian cherry or dogwood, has long been cultivated in many different countries [1]. It is one of the most valuable fruit plants from the dogwood family (*Cornaceae*). Moreover, Cornelian cherry is a well-recognized food ingredient in the traditional cuisines of Poland, the Czech Republic, Serbia, Romania, Turkey, and Iran. It is classified as a slow-growing and long-lived plant. This melliferous plant blooms very early, before the appearance of leaves. Ripe fruits are harvested from mid-August to the end of October. The fruit of the dogwood tree is a spherical or elliptical drupe. In recent years, many researchers have started to pay special attention to dogwood fruits and describe their properties, not only their taste and quality, but also their health-promoting effects. Furthermore, dogwood is a valuable plant from the point of view of pharmacology and the food industry and is often processed to produce tinctures, tonics, drinks, syrups, and jams [2]. Due to its high content of antioxidants, it could also be an attractive material for the cosmetic industry. Currently, it is generally accepted that *C. mas* L. is a rich source of vitamin C and polyphenols [3]. Moreover, substantial amounts of flavonoids, anthocyanins, and iridoids have been identified in the fruits and leaves of the cornelian cherry [4]. The high content of vitamins, polyphenols, and antioxidants results in strong antioxidant properties of *C. mas* L. fruits and their extracts [5]. These compounds are linked with high radical scavenging properties, antitumor effects, and anti-inflammatory activity [6]. The health-benefit properties of *C. mas* L. are ascribed to the induction of the expression of antioxidant enzymes such as superoxide dismutase 1 (SOD1). Given these properties, *C. mas* L. extracts have been used in, e.g., dermatology and cosmetology [7]. Additionally, some studies (e.g., Klymenko et al. and Popović et al.) have shown that different species of *Cornus* may exhibit distinct antioxidant properties and different contents of terpenoids and anthocyanins, which indicates that these parameters in specific species or even varieties of *Cornus* need to be determined [8,9]. Peroxisome proliferator-activated receptor gamma (PPARγ) is a nuclear receptor that regulates the expression of genes involved in insulin sensitivity, adipogenesis, glucose homeostasis, cell differentiation, inflammation, and cell death [10]. Moreover, it is known that SOD1 expression can be controlled by PPARγ [11], which confirms its important role in controlling the expression of antioxidant enzymes. To date, PPARγ agonists (rosiglitazone and pioglitazone) have been shown to increase insulin sensitivity and responsiveness, correct hyperglycemia, hyperinsulinemia, and hypertriglyceridemia in type 2 diabetes mellitus (DM2) patients, enhance adipocyte differentiation, and increase the mRNA expression of insulin-responsive *glucose transporter 4 (GLUT4)* in DM2 muscle tissue [12]. Moreover, through transrepression, PPARγ may exhibit an anti-inflammatory action by interfering with the nuclear factor kappa-light-chain-enhancer of the activated B (NF-κB) pathway in a DNA-independent manner [13].

Previous studies have indicated that extracts from *C. mas* L. fruits increased *PPARα* and *PPARγ* mRNA expression in rabbit aorta [14]. Moreover, the administration of a cornelian cherry extract resulted in a decrease in triglyceride, leptin, and resistin levels and an increase in adiponectin levels. These results suggest the potential value of cornelian cherry extracts in mitigating the risk of the development and the intensity of symptoms of obesity-related cardiovascular diseases and metabolic disorders [14,15]. Moreover, the anti-inflammatory effect of a *C. mas* L. fruit extract was observed in the paw tissue of Wistar rats [16], where the extract was found to significantly suppress the production of interleukin 1β (IL1β) and interleukin 13 (IL-13) and enhance the production of interleukin 10 (IL-10) in the paw tissue after induction of inflammation [16]. Additionally, the cornelian cherry extract led to a significant reduction in the mRNA expression of matrix metalloproteinase-1 (MMP-1), interleukin 6 (IL-6), and nicotinamide adenine dinucleotide phosphate (NADPH) oxidases (NOX) in the aorta and a decrease in vascular cell adhesion molecule-1 (VCAM-1), intercellular adhesion molecule-1 (ICAM-1), paraoxonase-1 (PON-1), and procalcitonin (PCT) serum levels in male New Zealand rabbits [17].

It is well-known that the composition of active substances and the mechanism of action of extracts depend not only on the plant species but also on its variety [18]. Therefore, the aim of this study was to compare the biological activities of three Ukrainian cultivars of *C. mas* L. with different fruit colors growing in the Polish region of Kielnarowa. To this end, biologically active compounds were chromatographically determined and the antioxidant and cytoprotective properties of water and ethanol extracts from the fruits of the tested dogwood cultivars were compared. Moreover, certain intracellular pathways (antioxidant-, PPARγ/PGC-1α-, and inflammation-related) were identified after treatment with extracts from specific cultivars of *C. mas* L.

## 2. Results

### 2.1. Chromatographic Analysis of Extracts from Fruit of C. mas L.

The phytochemical compositions of yellow, red, and dark red fruit extracts were investigated through ultra-performance liquid chromatography coupled with DAD and mass detectors (UPLC-DAD-MS). Table 1 provides the MS data of the main identified compounds. The results from a quantitative analysis, presented as µg/mL of the extracts, can be found in Table 2. The identification was based on a comparison of MS and UV/Vis spectra with standards or components that were tentatively identified based on literature data [19,20].

The obtained profiles were similar to those found in the literature [20,21]. The most abundant compound was the iridoid, loganic acid (*m/z* = 375.13), followed by derivatives of gallic acid, namely, galloyl hexoside and galloyl-d-sedoheptulose, with a common fragment *m/z* = 169, which is typical for gallic acid.

The results of our chromatographic analysis indicated statistically significant differences in the contents of most of the identified phytochemicals among the individual dogwood extracts. No significant differences were observed only in the case of quinic acid. These differences were noted both between the individual types of extracts and between extracts obtained from dogwood fruits of different colors. The loganic acid content varied from 41.09 ± 1.56 µg/mL (YE) to 72.45 ± 2.14 µg/mL in DRE. The contents of galloyl hexoside and galloyl-d-sedoheptulose were in the range of 11.94–20.69 and 10.60–19.78 µg/mL, with the highest content of these compounds in DRE. Moreover, the dark red fruit aqueous ethanol extract exhibited the highest content of phenolic (quinic, gallic, ellagic) acids among all the extracts tested. 

The most significant difference between the samples was the absence of anthocyanins, i.e., cyanidin 3-*O*-galactoside and pelargonidin 3-*O*-glucoside, in the extract from the yellow fruits. 

### 2.2. Determination of Antioxidant Properties

Synthetic free radicals DPPH and ABTS were used to evaluate the antioxidant properties of the individual *C. mas* L. extracts. The results are presented as the value of the IC_50_ parameter, which defines the antioxidant concentration causing a 50% decrease in the initial concentration of DPPH and ABTS^+^ radicals. The measurements were performed in a concentration range from 10 to 2000 µg/mL, and the results are presented in Table 3.

As shown, DRE exhibited the best radical scavenging properties, reaching an IC_50_ value of 572.24 ± 3.56 µg/mL in the DPPH assay and 541.50 ± 3.97 µg/mL in the ABTS assay. In comparing the type of extractants, it was found that the ethanolic extracts from all three cultivars of *C. mas* L. had the most favorable antioxidant properties. 

### 2.3. Protein Expression

**Figure 1:**
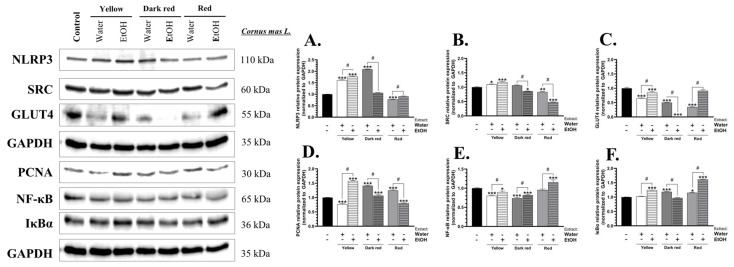
Protein expression of NLRP3 (**A**), SRC (**B**), GLUT4 (**C**), PCNA (**D**), NF-κB (**E**), and IκBα (**F**) in BJ cells after treatment with 250 µg/mL of water and ethanol extracts of yellow fruit, dark red fruit, and red fruit *C. mas* L. Means ± SD denoted as *, **, and *** are statistically different at *p* < 0.05, *p* < 0.01, and *p* < 0.001, compared to the control, respectively (ANOVA, Tukey’s post hoc test), while data marked as # indicate statistical differences between certain groups at *p* < 0.05.

The nucleotide-binding domain, leucine-containing family, and pyrin domain-containing-3 (NLRP3) protein expression was increased by 61.35, 77.26, and 108.99%, respectively, in BJ cells treated with the water and ethanol extracts of yellow fruit *C. mas* L. and with the water extract of dark red fruit *C. mas* L., compared to the control (Figure 1A). In turn, the protein expression of this protein was decreased by 22.69% only in cells treated with the water extract of red fruit *C. mas* L., compared to the control (Figure 1A).

The proto-oncogene tyrosine-protein kinase (SRC) protein expression was increased by 9.89 and 17.95% in cells treated with the water and ethanol extracts of yellow fruit *C. mas* L., respectively, compared to the control (Figure 1B). In contrast, the BJ cells treated with the ethanol extract of dark red fruit *C. mas* L. and the water and ethanol extracts of red fruit *C. mas* L. were characterized by a decrease in the SRC protein expression by 13.93, 16.82, and 51.71%, respectively, compared to the control (Figure 1B).

The GLUT4 protein expression was decreased significantly by 34.89, 14.33, 49.94, 98.42, and 65.37% in cells treated with the water and ethanol extracts of yellow fruit *C. mas* L., the water and ethanol extracts of dark red fruit *C. mas* L., and the water extract of red fruit *C. mas* L., respectively, compared to the control (Figure 1C). 

The protein expression of proliferating cell nuclear antigen (PCNA) decreased by 23.29 and 19.72% in cells treated with the water extract of yellow fruit *C. mas* L. and the ethanol extract of red fruit *C. mas* L., respectively, compared to the control (Figure 1D). In turn, a 57.39, 41.07, 6.84, and 24.70% increase in the expression of this protein was observed in BJ cells treated with the ethanol extract of yellow fruit *C. mas* L., the water and ethanol extracts of dark red fruit *C. mas* L., and the water extract of red fruit *C. mas* L., respectively, compared to the control (Figure 1D).

The NF-κB protein expression was increased by 15.66% only in cells treated with the ethanol extract of red fruit *C. mas* L., compared to the control (Figure 1E). In contrast, the water and ethanol extracts of yellow fruit *C. mas* L. and the water and ethanol extracts of dark red fruit *C. mas* L. caused a 19.85, 10.29, 26.29, and 18.16% decrease in NF-κB protein expression, respectively, compared to the control (Figure 1E). 

The protein expression of the nuclear factor of kappa light polypeptide gene enhancer in B-cells inhibitor alpha (IκBα) was increased by 24.39, 18.45, 15.20, and 61.34%, compared to the control, respectively, in BJ cells treated with the ethanol extract of yellow fruit *C. mas* L., the water extract of dark red fruit *C. mas* L., and the water and ethanol extracts of red fruit *C. mas* L. (Figure 1F). 

**Figure 2:**
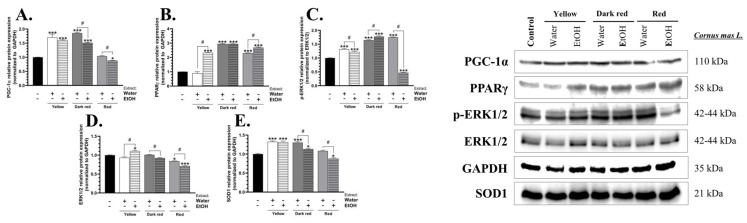
Protein expression of PGC-1α (**A**), PPARγ (**B**), p-ERK1/2 (**C**), ERK1/2 (**D**), and SOD1 (**E**) in BJ cells after treatment with 250 µg/mL of water and ethanol extracts of yellow fruit, dark red fruit, and red fruit *C. mas* L. Means ± SD denoted as *, and *** are statistically different at *p* < 0.05, and *p* < 0.001, compared to the control, respectively (ANOVA, Tukey’s post-hoc test), while data marked as # indicate statistical differences between certain groups at *p* < 0.05.

The peroxisome proliferator-activated receptor-gamma coactivator-1 alpha (PGC-1α) protein expression was increased by 70.33, 60.03, 85.56, and 51.33% in cells treated with the water and ethanol extracts of yellow-fruit *C. mas* L. and the water and ethanol extracts of dark red fruit *C. mas* L., respectively, compared to the control (Figure 2A). In turn, the ethanol extract of red fruit *C. mas* L. caused an 11.95% decrease in the expression of this protein, compared to the control (Figure 2A).

The PPARγ protein expression increased by 133.01, 193.38, 193.84, 130.58, and 168.43% after treatment of the cells with the ethanol extract of yellow fruit *C. mas* L., the water and ethanol extracts of dark red fruit *C. mas* L., and the water and ethanol extracts of red fruit *C. mas* L., respectively, compared to the control (Figure 2B).

The phosphorylation level of extracellular signal-regulated protein kinase 1 and 2 (ERK1/2) proteins was increased by 31.52, 21.89, 64.63, 77.65, and 74.89% in the BJ cells treated with the water and ethanol extracts of yellow fruit *C. mas* L., the water and ethanol extracts of dark red-fruit *C. mas* L., and the water extract of red fruit *C. mas* L., compared to the control, respectively (Figure 2C). In turn, a 52.79% decrease in the phosphorylation level of this protein was observed in cells treated with the ethanol extract of red fruit *C. mas* L., compared to the control (Figure 2C).

The ERK1/2 protein expression was increased by 10.63% only in cells treated with the ethanol extract of yellow fruit *C. mas* L., compared to the control (Figure 2D). In contrast, a 15.88 and 28.93% decrease in this parameter was shown in the BJ cells treated with the water and ethanol extracts of red fruit *C. mas* L., respectively, compared to the control (Figure 2D).

The superoxide dismutase 1 (SOD1) protein expression increased by 32.39, 31.23, 30.35, and 13.01% after the treatment of the cells with the water and ethanol extracts of yellow fruit *C. mas* L. and the water and ethanol extracts of dark red fruit *C. mas* L., respectively, compared to the control (Figure 2E). In turn, the treatment of the cells with the ethanol extract of red fruit *C. mas* L. caused an 11.86% decrease in the SOD1 protein expression, compared to the control (Figure 2E).

### 2.4. Cytotoxicity Analysis Using the Alamar Blue (AB) Assay

**Figure 3:**
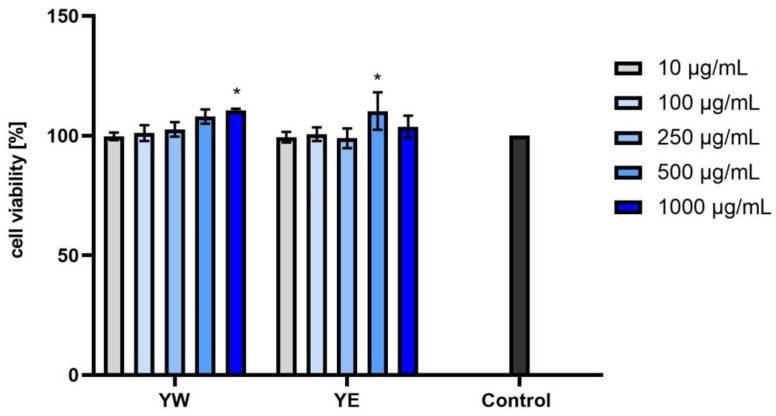
Effect of yellow fruit *C. mas* L. extracts (water (YW) and water–ethanol (YE)) on the reduction of resazurin in cultured fibroblasts after 24 h of exposure. Data are the means ± SD of three independent experiments, each consisting of three replicates per test group. * *p* < 0.01.

**Figure 4:**
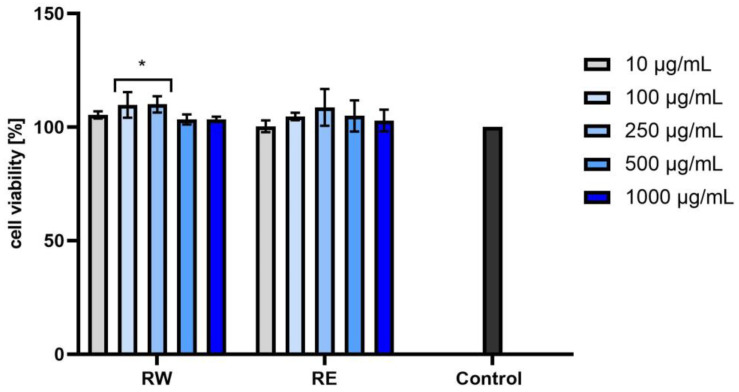
Effect of red fruit *C. mas* L. extracts (water (YW) and water–ethanol (YE)) on the reduction of resazurin in cultured fibroblasts after 24 h of exposure. Data are the means ± SD of three independent experiments, each consisting of three replicates per test group. * *p* < 0.01.

**Figure 5:**
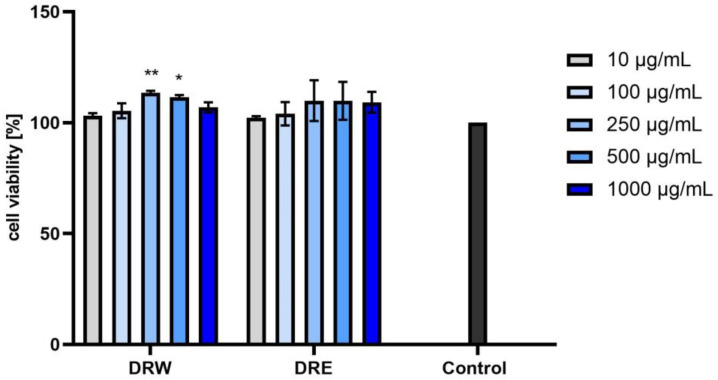
Effect of dark red fruit *C. mas* L. extracts (water (YW) and water–ethanol (YE)) on the reduction of resazurin in cultured fibroblasts after 24 h of exposure. Data are the means ± SD of three independent experiments, each consisting of three replicates per test group. ** *p* < 0.001, * *p* < 0.01.

Our analyses showed no cytotoxicity of the tested extracts toward normal skin fibroblasts in vitro. As shown in Figure 3, Figure 4 and Figure 5, the effect depended on both the dose and the type of extract used. Slight differences in activity were observed between the water and water–ethanolic extracts and between the individual dogwood cultivars. Taking into account these results, it can be concluded that the strongest pro-proliferative effect of the three tested cultivars was exerted by the BBW extract. Statistically significant differences were observed when the extracts were tested at concentrations of 250, 500, or 1000 µg/mL. The aqueous and aqueous–ethanol yellow fruit extract showed a proliferative capacity of 110.54% ± 1.58 at a concentration of 1000 µg/mL and 110.26% ± 7.79 at a concentration of 500 µg/mL. The most favorable results of cell viability assessed for the red fruit and dark red fruit extracts were obtained at a concentration of 250 µg/mL, i.e., 110% ± 3.60 and 108.66% ± 8.08 for the aqueous and aqueous–ethanol extracts, respectively. In the case of the dark red fruit extract, the proliferation values were 113.50% ± 0.84 and 109.93% ± 9.20 for the aqueous and aqueous–ethanol extract, respectively. The results were compared to the control (cells not treated with the extracts), shown as 100% of cell viability. However, the differences between the individual extracts were small, and all the six analyzed extracts had a positive effect on the metabolic activity in the tested cell line.

## 3. Discussion

The pro-health effects of dogwood fruit extracts are the subject of research carried out by many scientific groups. This effect is closely related to the high content of biologically active compounds in these fruits. Extracts obtained from these fruits are a valuable source of phenolic compounds, flavonoids, vitamin C, iridoids, organic acids, anthocyanins, and mono- and disaccharides, and their antioxidant activity depends primarily on the number and position of hydroxyl groups in the molecular structure [16,22,23].

The main antioxidant components of dogwood fruit are phenolic compounds, and their total content is proportional to the antioxidant activity. These compounds primarily include chlorogenic acid, caffeic acid, quercetin, (+)-catechin, (−)-epicatechin, and ellagic acid [16]. Their content depends on various factors, e.g., the variety of the plant or the year of harvest. Their average content is estimated at approximately 370 mg/g fresh weight [24,25]. The content of health-promoting compounds is also significantly influenced by the method of extraction of the plant material. Research on the optimization of the extraction of active compounds from plant material has shown that the applied microwave- and ultrasonic-based techniques are characterized by much greater extraction efficiency than the traditional maceration method. At the same time, it was found that changing the polarity of the solvent by adding ethyl alcohol significantly increases the amount of the extracted compounds [26,27]. Similar conclusions were formulated in the present study.

The ability of the analyzed dogwood extracts to scavenge free radicals is undoubtedly related to the action of biologically active compounds present in the fruits of this plant. Various antioxidant compounds, whose presence was confirmed during chromatographic analyses of the tested *C. mas* L. extracts, may be responsible for this effect. The neutralization of free radicals may be the result of the action of various phenolic acids that were detected in the tested extracts, such as gallic acid, loganic acid, ellagic acid, or chlorogenic acid. These compounds have proven antioxidant properties and the ability to inhibit cell damage caused by free radicals and suppress specific enzymes engaged in intracellular ROS overproduction [28,29]. Literature data indicate that phenolic compounds can inhibit the spread of lipid oxidation by intercepting intramembrane radicals and increasing the fluidity of the cell membrane. Moreover, these phytochemicals have the ability to disorganize lipid chains, which consequently results in the inhibition of the propagation of free radicals [30,31,32]. The antioxidant effect of dogwood is probably also related to the action of anthocyanins, which neutralize free radicals effectively and thus prevent oxidative stress. The antioxidant properties of this group of chemical compounds are enhanced by the presence of hydroxyl groups in the rings, which can chelate metal ions, and by the arylation of sugar residues by phenolic acids [33]. Anthocyanins also have the ability to inhibit lipid peroxidation and fatty acid autoxidation by acting on suboxides and hydrogen radicals and chelating anions of various metals [33]. The antioxidant potential of the tested extracts may also be the result of the action of iridoids, mainly loganic acid, which can exert antioxidant effects indirectly by stimulating the antioxidant defense system and directly by removing ROS [34,35].

The mechanism of the antioxidant action of phenolic acids and other phytochemicals with antioxidant activity may be based on the transfer of hydrogen atoms and single electrons and the ability to chelate transition metals [36]. Therefore, the antioxidant properties of dogwood fruit have been confirmed by other authors using methods other than DPPH or ABTS, such as FRAP or assessment of the ability to chelate iron ions [37,38]. As reported by many authors, the strong antioxidant properties of this plant may be the result of the action of various antioxidant mechanisms, which, together, are responsible for the extraordinary antioxidant potential of dogwood fruits. Literature data also indicate variability in the antioxidant activity of dogwood fruits depending on the examined variety, genotype, or growing conditions [9]. This is primarily related to differences in the content of biologically active compounds in the fruits of different genotypes of this plant, which was also demonstrated in this study. The present results indicate the lowest content of biologically active compounds in the yellow fruits, which correlates with the lower antioxidant activity of this cultivar (measured in situ). This is probably also related to the absence of anthocyanins, which occur in red and dark red fruits and are responsible for their greater antioxidant potential. As revealed by our analysis, dogwood cultivars with red and dark red fruits are characterized by the presence of a wide spectrum of plant phytochemicals and have stronger biological effects than cultivars with yellow fruits.

Aurori et al. reported a protective effect of dogwood fruits (at a dose of 40 mg/kg) on renal tubules in studies on Wistar rats [39]. In their study on kidney epithelial cells (Vero), Yarim et al. also showed that a cornelian fruit extract (in a concentration range of 1–100 μg/mL) protected cells against in vitro oxidative damage caused by cisplatin [40]. Tiptiri-Kourpeti et al. showed that dogwood fruit juice had an antiproliferative effect (in a concentration range of 0.01–1.0% *v*/*v*) on four human cancer cell lines, i.e., MCF-7 (breast adenocarcinoma), HepG2 (hepatocellular carcinoma), and Caco2 and HT-29 (colon adenocarcinoma). They observed similar effects in CT26 mouse colon cancer cells. However, those authors did not observe any inhibition of tumor growth in an in vivo experimental mouse model of colon cancer [41]. Extracts from dogwood plants are often perceived as valuable raw materials with a positive effect on skin cells. Biologically active compounds contained in dogwood fruits increase the activity of antioxidant enzymes and support the work of repair enzymes. Moreover, active compounds present in dogwood fruits inhibit enzymes such as hyaluronidase, elastase, and collagenase, thus limiting the degradation of hyaluronic acid and collagen and elastin fibers [7,42]. Additionally, dogwood fruit extracts can stimulate cell migration, contributing to faster wound healing, increasing skin hydration, and limiting transepidermal water loss [7].

Our data show that the studied extracts of *C. mas* L. increase PPARγ and PGC-1α protein expression, which confirms the activation of the PPARγ-dependent molecular pathway. Moreover, both PPARγ and PCG-1α are involved in the activation of the expression of antioxidant enzymes, such as SOD or catalase (CAT) [43]. Our data, which show that the extracts increased SOD1 protein expression, are in line with the mechanism of action of the PPARγ/PGC-1α molecular pathway. SRC kinase is needed for PPARγ ligand signal transduction and for PGC-1α dimerization with PPARγ, which was also confirmed by our western blot analyses [44]. Danielewski et al. noted that extracts from *C. mas* L. fruits increased *PPARα* and *PPARγ* mRNA expression in rabbit aorta [14]. Therefore, our data are consistent with the current state of knowledge and confirm the discoveries reported by Danielewski et al. [15]. Interestingly, the studied extracts decreased the content of GLUT4 protein, which is one of the transmembrane glucose transporters. To date, it has been reported that PPARγ agonists increase *GLUT4* mRNA expression in DM2 muscle tissue [12]. On the other hand, it has been reported that, in primary rat adipocytes and CHO-K1 cells, PPARγ agonists repress GLUT4 promoter activity and protein expression via direct and specific binding of PPARγ/retinoid X receptor-α (RXRα) to the GLUT4 promoter [45]. These data show that the effect of PPARγ on GLUT4 expression is tissue-dependent; our data are in line with the current knowledge. It has been widely reported that PPARγ pathway activation through transrepression interferes with the NF-κB pathway [13]. This phenomenon was confirmed in our experimental model, in which almost all the studied extracts decreased the NF-κB protein expression in the BJ cell culture. The NRLP3 protein is mainly connected with inflammasome formation; however, the non-canonical pathway is associated with increased oxidative stress and the production of antioxidant enzymes [46]. It is worth noting that after the treatment of the cells with the extracts in our experiments, the highest NRLP3 protein expression correlated with the highest expression of the SOD1 protein, which suggests the activation of an antioxidant response. We believe that this is a result of the non-canonical role of the NRLP3 protein. In our model, increased PCNA expression was also observed in the groups with high expression of SOD1 and NRLP3, which is unusual. PCNA is a well-established marker of proliferating cells; however, PCNA protein expression can increase in response to ROS [47]. Similarly, the observed increase in the ERK1/2 kinase expression may have been a result of ROS overproduction [48]. Last but not least, it is worth mentioning that the extract from the red fruit *C. mas* L., especially the ethanol-based one, may act in a different way than the other extracts. These discrepancies may be related to the different chemical composition, as shown by our chromatographic analysis of the tested extracts. Notably, the red ethanol extract contained the highest level of chlorogenic acid and p-coumaroylquinic acid. It has been reported that p-coumaroylquinic acid can affect metabolic pathways that are dependent on PPARγ and PGC-1α [49], which explains our results. However, more research is needed to fully elucidate this mechanism.

## 4. Materials and Methods

### 4.1. Reagents

In our study, 2,2-Azino-bis-3-ethylbenzothiazoline-6-sulfonic acid (ABTS solution), 2,2-diphenyl-1-picrylhydrazyl (DPPH), N,N,N′,N′-tetramethylethylenediamine (TEMED), acrylamide/bisacrylamide, ammonium persulfate (APS), ascorbic acid (vitamin C), bovine serum albumin (BSA), ethanol (96%), glycine, methanol, Ponceau S, resazurin sodium salt (RES), sodium dodecyl sulfate (SDS), Trolox, trypsin-EDTA solution, Tris-HCl, Tris-Base, and Tween-20 were purchased from Merck KGaA (Darmstadt, Germany). The PVDF membrane with 0.45 µm-pore size and primary antibodies against SOD1 were obtained from Santa Cruz Biotechnology (Santa Cruz, CA, USA). The primary antibodies against GAPDH, NF-κB, IκBα, SRC, GLUT4, PCNA, PGC-1α, PPARγ, p-ERK1/2, and ERK1/2 were purchased from ABClonal (Woburn, MA, USA). The primary antibodies against NLRP3, anti-mouse- and anti-rabbit-HRP-conjugated antibodies were purchased from ThermoFisher (Waltham, MA, USA). Potassium persulfate (Warchem, Zakręt, Poland), antibiotics (Penicillin-Streptomycin, Life Technologies, Bleiswijk, The Netherlands), DMEM (Dulbecco’s Modification of Eagle’s Medium, Biological Industries, Beit Haemek, Israel), FBS (Fetal Bovine Serum, Biological Industries, Genos, Lodz, Poland), and phosphate buffered saline (PBS, pH 7.00 ± 0.05, ChemPur, Piekary Ślaskie, Poland) were also used during the analyses. 

### 4.2. Plant Material and Extraction Procedure

To prepare dogwood extracts, fresh fruits of three Ukrainian-origin cultivars of *C. mas* L., i.e., Jantarnyj (yellow fruit), Korralowyj Marka (red fruit), and Władimirskij (dark ruby red fruit), were used. The dogwood seedlings from Ukraine were cultivated in south-eastern Poland at a geographical latitude of 49.9501 degrees and a longitude of 22.0604 degrees (Kelnarowa). The extraction of active substances was performed two days after fruit harvest. The prepared extracts were stored as aliquots frozen at −80 °C if further analyses were not possible immediately. As part of the work, two types of extracts were prepared for each cultivar: aqueous and aqueous–ethanol (with a final ethanol concentration of 50% (*v/v*)). First, 5 g of fruit of each cultivar was added to a glass beaker and 100 mL of the extraction solution (water or a mixture of water and ethanol) was poured in. The solutions prepared in this way were stirred with a magnetic stirrer for 14 h at 22 °C. Then, the solutions were subjected to ultrasound in an ultrasonic bath (Digital Ultrasonic Cleaner—BS-UC10; Beauty System; Wrocław; Poland) for 30 min, according to the procedure described by Yang et al., with minor modifications [50]. The temperature of the solutions during extraction changed from 21.5 to 25.7 °C. The extracts prepared in this way were filtered using Whatman filter paper no. 10 (Thermo Fisher Scientific, Gothenburg, Sweden) and evaporated using a vacuum evaporator (Eppendorf Concentrator plus™; Eppendorf, Hamburg, Germany). After evaporation, the concentrated extracts were dissolved in a sterile PBS solution to obtain a solution with a concentration of 10 mg/mL.

### 4.3. Chromatographic Determination of Biologically Active Compounds

All standard compounds and reagents, such as MS-grade formic acid and MS-grade acetonitrile, were obtained from Sigma-Aldrich (St. Louis, MO, USA). Deionized water was acquired using the Ultrapure Millipore Direct-Q^®^ 3UV-R system (Merck KGaA, Darmstadt, Germany).

Chromatographic separation was achieved using an ultra-high-performance liquid chromatographic system (UHPLC), specifically, the Infinity Series II equipped with a DAD detector and an Agilent 6224 ESI/TOF mass detector (Agilent Technologies, Santa Clara, CA, USA). The separation was performed on a Titan RP18 column (Supelco, Sigma-Aldrich, Burlington, MA, USA) with dimensions of 10 cm × 2.1 mm i.d. and a particle size of 1.9 µm. The mobile phase comprised two components: water containing 0.05% formic acid (solvent A) and acetonitrile containing 0.05% formic acid (solvent B), delivered at a flow rate of 0.2 mL/min. The gradient program employed was as follows: 0–8 min transitioning from 98% A to 93% A; 8–20 min maintaining 93% A; 20–40 min transitioning from 93% A to 80% A; and 40–60 min changing from 80% A to 70% A. The thermostat was set at 30 °C. UV/VIS spectra were recorded in the range from 200 to 600 nm. The parameters of mass spectrometry with electrospray ionization (MS-ESI) included a drying gas temperature of 325 °C, a drying gas flow rate of 8 L/min, a nebulizer pressure of 30 psi, a capillary voltage of 3500 V, a skimmer voltage of 65 V, and a fragmentor voltage of 220 V. Negative ionization mode was employed to acquire ions within a mass range from 100 to 1000 *m/z* [5]. Base peak chromatogram (BPC) in Supplementary Materials (Figure S1).

### 4.4. Determination of RADICAL Scavenging Properties

#### 4.4.1. DPPH (1,1-Diphenyl-2-picrylhydrazyl) Radical Scavenging Assay

The antioxidant properties of six tested extracts from *C. mas* L. were assessed using the DPPH radical, following the procedure described by Miller et al. [51]. The reduction of the DPPH radical by the analyzed samples was measured in a concentration range of 10–2000 µg/mL. For this purpose, 100 µL of a 4 mM methanolic DPPH solution (Merck KGaA, Darmstadt, Germany) and 100 µL of the tested samples were added to each well in a 96-well plate, and the solution was mixed thoroughly. In the negative control, distilled water was used instead of the extract. Vitamin C and Trolox at a concentration of 15.5 µg/mL were used as positive controls. The measurements of the absorbance of the tested samples were performed every 5 min for 20 min at a wavelength of 517 nm. A UV/VIS Filter Max spectrophotometer (Thermo Fisher Scientific, Waltham, MA, USA) was used to measure the absorbance of the tested samples. Three independent experiments were performed, during which each sample was tested in three repetitions. Then, using Equation (1), the percentage of DPPH radical scavenging by each individual dogwood sample was calculated. Then, a calibration curve was made, based on which the IC_50_ value was determined. Based on these values, the concentrations of the tested extracts which were able to reduce the initial concentration of the DPPH radical by 50% were determined.

(1) $$\%\;DPPH\;scavenging=\frac{Abs\;control-Abs\;sample}{Abs\;control}\times 100$$

Abs sample—absorbance of the sample; Abs control—absorbance of the control sample.

#### 4.4.2. ABTS Scavenging Assay

The antioxidant properties were also assessed using the ABTS radical and the methodology described by Miller et al. [51]. Initially, a mixture of 7 mM ABTS solution (Merck KGaA, Darmstadt, Germany) and 2.4 mM potassium persulfate (Warchem, Zakręt, Poland) was prepared in a 1:1 ratio. This mixture was left in the dark at room temperature for 16 h. After this time, the solution was diluted with methanol until it reached an absorbance of 1.0 ± 0.05 at a wavelength of 734 nm. The ABTS solution prepared in this way was mixed in a 1:1 ratio with individual concentrations of the tested dogwood extracts (in the concentration range of 10–2000 µg/mL). Then, the absorbance of the tested samples was measured at a wavelength of 734 nm using a UV/VIS spectrophotometer (Aquamate Helton). In the negative control sample, methanol was added instead of the extract solution. Vitamin C and Trolox at a concentration of 15.5 µg/mL were used as positive controls. Each test sample was performed in triplicate. The scavenging of the ABTS radical by the tested samples was calculated using Equation (2). A calibration curve was also prepared on the basis of which the IC_50_ was determined, which allowed us to determine the concentrations of the individual extracts that caused a 50% decrease in the initial concentration of the ABTS radical.

(2) $$\%\;of\;ABTS•+scavenging=1-\frac{Abs\;sample}{Abs\;control}\times 100$$

Abs sample—absorbance of the sample; Abs control—absorbance of the control sample.

### 4.5. Western Blot

Our analysis was performed following the procedure described in a previous report, with minor modifications [52,53]. In brief, BJ cells were seeded on 6-well plates at a density of 2.2 × 10^5^ cells/well 24 h before the experiment. After this interval, the medium was removed, and fresh medium containing 250 µg/mL of the tested extracts was added to the cells. After 24 h, the cells were lysed and scraped using a radioimmunoassay buffer (RIPA) and stored at −80 °C until analysis. Shortly before the assay, the samples were thawed on ice, and the protein concentration was assessed with the BCA method, followed by standardization of the protein content in each sample and denaturation in the presence of 5× Laemmle buffer [54]. Forty micrograms of the sample were loaded and separated on a 7.5%-acrylamide/bisacrylamide gel (SDS-PAGE electrophoresis) using the Mini-PROTEAN Tetra Cell (Bio-Rad, Hercules, CA, USA). Subsequently, electrotransfer on a PVDF membrane was performed at 35 V at 4 °C overnight using Mini Trans-Blot^®^ (Bio-Rad). After this time, non-specific sites were blocked using 1% of BSA in TBST, followed by overnight incubation of the membranes with primary antibodies (the dilution, catalog numbers, and producers are specified in Table 4). Next, the membranes were washed three times with TBST for 10 min and secondary anti-mouse or anti-rabbit HRP-conjugated antibodies were added for 1 h at RT. Subsequently, the membranes were washed three times with TBST and once with TBS, followed by chemiluminescence-based detection (LiCor C-DiGit, Lincoln, NE, USA). The density of the bands was measured using GelQuant.NET version 1.8.2. free software (provided by biochemlabsolutions.com, accessed on 10 January 2024). The GAPDG protein expression was always used as a loading control after stripping the membranes with Mild Stripping Buffer (pH = 2.2, 0.1% of SDS, 1.5% of glycine, 1% of Tween-20).

### 4.6. Cytotoxicity Analysis

#### 4.6.1. Cell Culture

Cytotoxicity analyses were performed on BJ human fibroblasts (ATCC^®^ CRL-2522™; American Type Culture Collection, Manassas, VA, USA). The cells were grown in culture flasks at 37 °C in an incubator in a humidified atmosphere containing 95% air and 5% carbon dioxide (CO_2_). During cultivation, DMEM medium (Dulbecco’s Modified Essential Medium; Biological Industries, Kibbutz Beit-Haemek, Israel) with the addition of L-glutamine (Merck KGaA, Darmstadt, Germany), 10% (*v/v*) FBS (Fetal Bovine Serum, Merck KGaA, Darmstadt, Germany), and 1% (*v/v*) antibiotic (100 U/mL penicillin and 1000 µg/mL streptomycin, Merck KGaA, Darmstadt, Germany) was used. For cytotoxicity analyses, the cells were transferred to 96-well plates at a density of 1 × 10^4^ cells/well and incubated for 24 h.

#### 4.6.2. Alamar Blue (AB) Assay

The cytotoxicity of the tested dogwood extracts was assessed using the Alamar Blue test according to the procedure described by Page et al., with modifications [55]. Briefly, the fibroblasts were incubated with the tested extracts at concentrations of 10, 100, 250, 500, and 1000 µg/mL for 24 h. Individual concentrations of the tested samples were prepared in DMEM medium. After incubation, the extract solutions were aspirated and a resazurin solution (Merck KGaA, Darmstadt, Germany) at a concentration of 60 μM was added to each well. Cells that were not exposed to the extracts and cultured in DMEM were used as the control. The plates were incubated with AB dye for 2 h. After this time, fluorescence was measured at a wavelength of 570 nm using a UV/VIS Filter Max spectrophotometer (Thermo Fisher Scientific, Waltham, MA, USA). As part of the analyses, three independent experiments were carried out, in which each extract concentration was tested in three repetitions.

### 4.7. Statistical Analysis

The values obtained during the experiments are presented as means ± standard deviations (SDs). As part of the statistical analysis, analysis of variance (ANOVA) and Dunnett’s intergroup post hoc test were performed. Statistical significance was determined at **** *p* < 0.0001, *** *p* < 0.001, ** *p* < 0.01, and * *p* < 0.05, compared with the controls. The statistical analyses were performed using GraphPad Prism 8.4.3 (GraphPad Software, Inc., San Diego, CA, USA). Data denoted as # were statistically different between each other at *p* < 0.05.

## 5. Conclusions

The results presented in this work indicate differences in the biological activity of extracts from dogwood cultivars with different fruit colors. Our data show that the studied extracts activate the PPARγ/PGC-1α molecular pathway in BJ cells and, through this mechanism, initiate antioxidant response. The absence of cytotoxicity toward skin fibroblasts and the multidirectional biological action of *C. mas* L. extracts indicate the potential use of extracts from this plant as ingredients of supplements and cosmetic preparations that help maintain good skin condition. Our study suggests the need for appropriate selection of the cultivar during the production stage to obtain products with the most satisfactory effects. Despite the health-promoting properties of the studied extracts, it is worth noting that extracts from each cultivar differ significantly and their composition may also depend on the place of plant growth. More research is needed to address this issue.

## Figures and Tables

**Table 1:** Mass data from a qualitative analysis of extracts from *C. mas* L. fruit. *—identification confirmed by standard.

Rt (min)	Observed Ion Mass [M-H]-/(Fragments)	Δ ppm	Formula	Identified
1.59	191.05657	2.39	C_7_H_12_O_6_	Quinic acid *
1.64	133.01485	4.50	C_4_H_6_O_5_	Malic acid *
2.04	191.01997	1.27	C_6_H_8_O_7_	Citric acid *
2.94	331.06755 (169)	1.44	C_13_H_16_O_10_	galloyl hexoside
3.49	169.01451 (125)	1.55	C_7_H_6_O_5_	Gallic acid *
3.89	361.07795 (271)	0.87	C_14_H_18_O_11_	Galloyl-d-sedoheptulose
6.12	153.01987	3.49	C_7_H_6_O_4_	Protocatechuic acid *
7.63	311.04099 (179, 149, 135)	0.43	C_13_H_12_O_9_	Caftaric acid * (cis/trans)
10.10	783.06895	0.39	C_34_H_24_O_22_	Oenothein C (Tannin)
11.50	375.13013	1.22	C_16_H_24_O_10_	Loganic acid *
11.71	311.04108 (179, 149, 135)	0.72	C_13_H_12_O_9_	Caftaric acid (cis/trans)
12.97	389.14599 (195, 345)	1.72	C_17_H_26_O_10_	Loganin
13.71	353.08843 (191, 179)	1.76	C_16_H_18_O_9_	Chlorogenic acid *
14.21	491.14124 (375)	1.24	C_20_H_28_O_14_	Loganic acid derivative
15.89	449.10923 (287)	0.66	C_21_H_22_O_11_	Aromadendrin hexoside
16.10	403.12532	1.82	C_17_H_24_O_11_	Secoxyloganin
16.46	447.09402 (285)	1.64	C_21_H_20_O_11_	Cyanidin 3-*O*-galactoside *
17.31	337.09214 (191, 173)	−2.22	C_16_H_18_O_8_	p-coumaroylquinic acid
17.76	431.09891 (269)	1.25	C_21_H_20_O_10_	Pelargonidin 3-*O*-glucoside
18.12	449.10968 (287)	1.65	C_21_H_22_O_11_	Aromadendrin hexoside
18.91	337.09374	2.51	C_16_H_18_O_8_	p-coumaroylquinic acid
18.81	403.12484	0.63	C_17_H_24_O_11_	Secoxyloganin
21.27	449.10977 (287)	1.85	C_21_H_22_O_11_	Aromadendrin hexoside
26.67	300.99929	0.99	C_14_H_6_O_8_	Ellagic acid *
27.54	463.08956 (301)	2.93	C_21_H_20_O_12_	Quercetin hexoside
27.96	477.06779 (301)	0.68	C_21_H_18_O_13_	Quercetin 3-glucuronide *
31.81	447.09501 (284)	3.85	C_21_H_20_O_11_	Kaempferol 3-*O*-galactoside
33.70	541.15619	−0.16	C_24_H_30_O_14_	Cornuside *

**Table 2:** Results of a quantitative analysis (presented as µg/mL ± standard deviations) of compounds identified in extracts obtained from *C. mas* L. fruit. Different letters on the same lines indicates a statistically significant difference (*p* < 0.05).

Compounds	YW [µg/mL]	YE [µg/mL]	RW [µg/mL]	RE [µg/mL]	DRW [µg/mL]	DRE [µg/mL]
Quinic acid *	15.64 ± 1.10 ^a^	15.34 ± 1.34 ^a^	16.62 ± 1.53 ^a^	16.45 ± 1.50 ^a^	15.69 ± 1.16 ^a^	16.32 ± 1.01 ^a^
Galloyl hexoside	6.71 ± 0.37 ^e^	11.93 ± 1.05 ^c^	9.38 ± 0.07 ^d^	16.77 ± 1.08 ^b^	11.94 ± 0.88 ^c^	20.69 ± 1.70 ^a^
Gallic acid *	1.65 ± 0.11 ^b^	3.44 ± 0.23 ^a^	1.89 ± 0.09 ^b^	2.91 ± 0.11 ^a^	3.08 ± 0.23 ^a^	3.37 ± 0.22 ^a^
Galloyl-d-sedoheptulose	4.96 ± 0.19 ^c^	10.60 ± 0.90 ^b^	9.92 ± 0.92 ^b^	17.73 ± 1.12 ^a^	16.99 ± 0.98 ^a^	19.78 ± 1.30 ^a^
Protocatechuic acid *	ND	ND	0.43 ± 0.03 ^c^	0.54 ± 0.05 ^b.c^	1.09 ± 0.08 ^a^	0.58 ± 0.04 ^b^
Caftaric acid * (cis/trans)	1.87 ± 0.16 ^b^	2.47 ± 0.02 ^a^	0.82 ± 0.04 ^c^	1.61 ± 0.11 ^b^	1.69 ± 0.13 ^b^	1.77 ± 0.12 ^b^
Loganic acid *	49.80 ± 4.80 ^c^	41.09 ± 1.56 ^d^	47.25 ± 2.25 ^c^	65.23 ± 3.58 ^b^	68.61 ± 1.58 ^a.b^	72.45 ± 2.14 ^a^
Loganin	0.15 ± 0.01 ^b^	0.14 ± 0.01 ^b^	0.12 ± 0.01 ^b^	0.32 ± 0.02 ^a^	0.34 ± 0.02 ^a^	0.33 ± 0.21 ^a^
Chlorogenic acid *	0.26 ± 0.01 ^e^	0.56 ± 0.05 ^c^	0.34 ± 0.03 ^d^	0.94 ± 0.08 ^a^	0.74 ± 0.07 ^b^	0.75 ± 0.63 ^a.b^
Cyanidin 3-*O*-galactoside *	ND	ND	0.94 ± 0.07 ^d^	2.46 ± 0.18 ^c^	3.90 ± 0.22 ^b^	5.62 ± 0.27 ^a^
p-coumaroylquinic acid	0.22 ± 0.02 ^d^	0.48 ± 0.02 ^c^	0.54 ± 0.03 ^c^	1.21 ± 0.10 ^a^	0.98 ± 0.06 ^b^	1.02 ± 0.10 ^a.b^
Pelargonidin 3-*O*-glucoside	ND	ND	1.47 ± 0.06 ^d^	5.52 ± 0.40 ^c^	7.67 ± 0.61 ^b^	10.14 ± 0.71 ^a^
Ellagic acid *	0.55 ± 0.04 ^d^	2.63 ± 0.14 ^c^	0.32 ± 0.02 ^e^	3.64 ± 0.24 ^b^	3.23 ± 0.19 ^b^	5.46 ± 0.33 ^a^
Quercetin 3-glucuronide *	2.71 ± 0.20 ^b^	2.98 ± 0.17 ^b^	1.87 ± 0.02 ^d^	2.35 ± 0.05 ^c^	4.28 ± 0.41 ^a^	4.42 ± 0.36 ^a^
Kaempferol 3-*O*-galactoside	ND	ND	0.14 ± 0.09 ^d^	0.36 ± 0.02 ^c^	0.55 ± 0.04 ^b^	0.77 ± 0.04 ^a^
Cornuside *	2.17 ± 0.18 ^c^	3.12 ± 0.09 ^a^	1.79 ± 0.09 ^d^	2.71 ± 0.22 ^b^	2.90 ± 0.14 ^a.b^	3.20 ± 0.13 ^a^

*—identification confirmed by standard; ND—not detected; YW—yellow fruit water extract; YE—yellow fruit ethanol extract; RW—red fruit water extract; RE—red fruit ethanol extract; DRW—dark ruby red water extract; DRE—dark ruby red ethanol extract.

**Table 3:** IC_50_ values for extracts from *C. mas* L. in the DPPH and ABTS radical assays. Different letters on the same lines indicates a statistically significant difference (*p* < 0.05).

Extract type	DPPH	ABTS
	IC_50_ ($\bar{x}$ ± SD, µg/mL)	IC_50_ ($\bar{x}$ ± SD, µg/mL)
Yellow-fruit water extract (YW)	885.69 ± 8.98 ^a^	1214.17 ± 9.41 ^a^
Yellow-fruit ethanol extract (YE)	645.62 ± 4.57 ^b^	862.73 ± 5.65 ^d^
Red-fruit water extract (RW)	637.28 ± 5.83 ^b^	1294.24 ± 10.65 ^b^
Red-fruit ethanol extract (RE)	619.76 ± 6.02 ^c^	653.96 ± 4.58 ^e^
Dark red-fruit water extract (DRW)	782.58 ± 5.82 ^d^	925.54 ± 7.64 ^c^
Dark red-fruit ethanol extract (DRE)	572.24 ± 3.56 ^e^	541.50 ± 3.97 ^f^

**Table 4:** Catalog numbers, producers, and dilutions of primary and secondary antibodies used in the study. Mo—mouse; Rb—rabbit; Go—goat.

Primary Antibodies			HRP-Conjugated Antibodies		
Target (Species)	Cat. Number/Producer	Diluted	Target (Species)	Cat. Number/Producer	Diluted
GAPDH (Mo)	AC033/ABClonal	1:100,000	anti-Mo-HRP-conjugated (Go)	31460/Thermo Fisher	1:2000
NF-κB (Mo)	A10609/ABClonal	1:2000			
SOD1 (Mo)	sc-101523/Santa Cruz Bt.	1:400			
NLRP3 (Rb)	PA5-79740/ThermoFisher	1:1000	anti-Rb-HRP-conjugated (Go)	31430/Thermo Fisher	1:2000
SRC (Rb)	A19119/ABClonal	1:4000			
GLUT4 (Rb)	A7637/ABClonal	1:1000			
PCNA (Rb)	A12427/ABClonal	1:2000			
IκBα (Rb)	A19714/ABClonal	1:2000			
PGC-1α (Rb)	A20995/ABClonal	1:2000			
PPARγ (Rb)	A11183/ABClonal	1:1500			
p-ERK1/2 (Rb)	AP0974/ABClonal	1:1000			
ERK1/2 (Rb)	A16686/ABClonal	1:2000			

## Data Availability

The data presented in this study are available on request from the corresponding author.

## Supplementary Materials

The supporting information can be downloaded at: https://www.mdpi.com/article/10.3390/ijms25105495/s1.
