# Supplementary material for: Comparison of the Antioxidant and Cytoprotective Properties of Extracts from Different Cultivars of Cornus mas L
Source: Int J Mol Sci. 2024 May 17;25(10):5495. doi: 10.3390/ijms25105495 (PMC11122231; doi:10.3390/ijms25105495)
Supplement: Supplementary file 1 [file ijms-25-05495-s001.zip › ijms-2962652-supplementary.pdf]

International Journal of *Molecular Sciences*

MDPI

Supplementary Materials

Comparison of the Antioxidant and Cytoprotective Properties of Extracts from Different Cultivars of *Cornus mas* L.

Tadeusz Pomianek [1], Martyna Zagórska-Dziok [2], Bartosz Skóra [3], Aleksandra Ziemlewska [2], Zofia Nizioł-Łukaszewska [2], Magdalena Wójciak [4], Ireneusz Sowa [4] and Konrad A. Szychowski [3,*]

[1] Department of Management, Faculty of Administration and Social Sciences, University of Information Technology and Management in Rzeszow, Sucharskiego 2, 35-225 Rzeszow, Poland; tpomianek@wsiz.edu.pl

[2] Department of Technology of Cosmetic and Pharmaceutical Products, Medical College, University of Information Technology and Management in Rzeszow, Sucharskiego 2, 35-225 Rzeszow, Poland; mzagorska@wsiz.edu.pl (M.Z.-D.); aziemlewska@wsiz.edu.pl (A.Z.); zniziol@wsiz.edu.pl (Z.N.-Ł.)

[3] Department of Biotechnology and Cell Biology, Medical College, University of Information Technology and Management in Rzeszow, Sucharskiego 2, 35-225 Rzeszow, Poland; bskora@wsiz.edu.pl

[4] Department of Analytical Chemistry, Medical University of Lublin, Aleje Raclawickie 1, 20-059 Lublin, Poland; magdalena.wojciak@umlub.pl (M.W.); ireneusz.sowa@umlub.pl (I.S.)

* Correspondence: kszychowski@wsiz.edu.pl

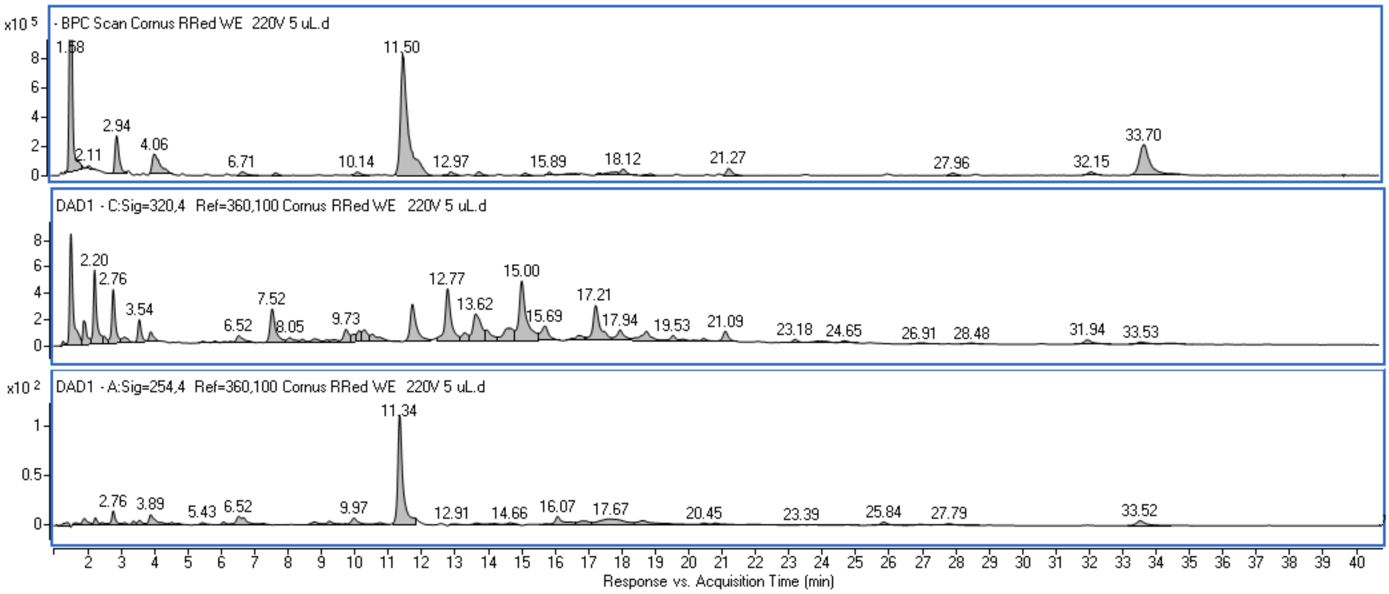

Figure S1. Base peak chromatogram (BPC) obtained in negative ionization mode and chromatograms registered at wavelengths of 320 and 254 nm od water extracts from fruits of *C. mas* L.
